# Impact of agitation/activation strategies on the antibiofilm potential of sodium hypochlorite/etidronate mixture in vitro

**DOI:** 10.1186/s12903-022-02222-1

**Published:** 2022-05-23

**Authors:** Ming Cai, Yanling Cai, Ruiqi Yang, Zhezhen Xu, Prasanna Neelakantan, Xi Wei

**Affiliations:** 1 Department of Operative Dentistry and Endodontics, Hospital of Stomatology, Sun Yat-Sen University, 56 Lingyuan Xi Road, Guangzhou, 510055 Guangdong China; 2 Guangdong Provincial Key Laboratory of Stomatology, Guangzhou, China; 3 Discipline of Endodontology, Faculty of Dentistry, The University of Hong Kong, The Prince Philip Dental Hospital, 34 Hospital Road, Sai Ying Pun, Pok Fu Lam, Hong Kong SAR China; 4 Department of General Dentistry, School of Stomatology, Kunming Medical University, Kunming, China

**Keywords:** Biofilm, Etidronate, Disinfection, Sodium hypochlorite, Ultrasonically activated irrigation, XP-endo Finisher, XP-endo Shaper

## Abstract

**Background:**

To investigate the effect of a rotary agitation method or ultrasonically activated irrigation on the antibiofilm effect of a mixture of sodium hypochlorite (NaOCl) and etidronate (1-hydroxyethylidene-1,1-bisphosphonate, HEBP) using a dual-species biofilm model in root canal system.

**Methods:**

Mature dual-species biofilms of Enterococcus faecalis and Streptococcus gordonii were formed in root canals of mandibular premolars. Teeth were randomly allotted (n = 12) to group 1, XP-endo Finisher (XPF); group 2, ultrasonically activated irrigation (UAI); group 3, syringe-and-needle irrigation (SNI). In all groups, canals were instrumented with a rotary instrument (XP-endo Shaper) prior to irrigant agitation/activation. A mixture containing 2.5% NaOCl and 9% HEBP was used throughout the experiment. Bacterial counts from the canal were determined using qPCR before preparation (S1), after preparation (S2), and after final irrigation agitation/activation (S3). Bacterial viability within the dentinal tubules in the coronal, middle and apical root-thirds was quantified using confocal microscopy after Live/Dead staining. The bacterial counts and viability were compared between groups using one-way ANOVA and post-hoc Tukey’s tests. Paired t-test was used to compare the bacterial counts within groups.

**Results:**

Instrumentation alone could significantly reduce the microbial counts in all the groups (P < 0.0001). Subsequent agitation/activation resulted in significant microbial reduction only in XPF and UAI (P < 0.05), both of which reduced significantly more microbial counts than SNI (P < 0.05). Live/Dead staining revealed that XPF and UAI showed significantly greater percentage of dead bacteria within the dentinal tubules than SNI in the coronal third (P < 0.05); UAI resulted in the significantly highest percentage of dead bacteria in the middle third (P < 0.05); while there was no significant difference between the groups in the apical third (P > 0.05).

**Conclusions:**

When using the sodium hypochlorite/etidronate mixture for irrigation, final irrigant agitation/activation with XP-endo Finisher or ultrasonic can improve disinfection of the main root canal space and the dentinal tubules in the coronal third, while ultrasonically activated irrigation appears to exhibit better disinfection within dentinal tubules in the middle third.

**Supplementary Information:**

The online version contains supplementary material available at 10.1186/s12903-022-02222-1.

## Background

The main goal of root canal debridement is to reduce the microbial load to a subcritical level which can facilitate periapical healing [1]. However, given that this critical threshold remains unknown, bacterial elimination and biofilm removal from root canals has been the focus of endodontic research for over a decade, albeit appearing to be an elusive goal [2]. Mechanical preparation of the root canal provides the shape and space for irrigation and obturation. Numerous instruments with innovative cross-sectional designs, alloy composition and kinematics are being introduced into clinical practice with the goals of less instrument separation, more contact with the root dentin walls and improvement of cleaning efficiency. Regardless of such innovations, 10–50% of the root canal walls remain untouched, depending on the instrument used [3]. Such untouched areas contain substantial amounts of microbial biofilms, pulp tissue remnants and hard tissue debris [4]. Thus, to maximize root canal disinfection, strategies of root canal debridement should be directed to use more effective irrigants and agitation/activation techniques.

Irrigants play a central role in disrupting biofilms and killing microbes that colonize in root [5]. Sodium hypochlorite (NaOCl) is considered the current gold standard of irrigants owing to its tissue dissolution and antimicrobial effects [5]. However, NaOCl is ineffective in removing the inorganic component of the smear layer and the accumulated hard tissue debris formed during preparation. So NaOCl was usually used in sequence with a chelator (ethylenediaminetetraacetic acid, EDTA). However, the active chlorine will be eradicated rapidly when NaOCl contacting with EDTA [6]. Furthermore, this sequence has harsh effects on root canal dentin [7]. Therefore, a mixture of NaOCl with a weak chelator (etidronate) was proposed to be used as an all-in-one solution during root canal preparation [8]. It was demonstrated that this mixture (NaOCl/HEBP) resulted in better removal of the smear layer [8] and biofilms [9, 10] compared to NaOCl/EDTA, with minimum detrimental effects on root canal dentine [7].

Multiple agitation/activation methods have been proposed to improve the efficacy of irrigants, including rotary instruments, oscillating instruments, sonics, ultrasonics, multi-sonics and lasers. While the evidence based on the efficacy of ultrasonically activated irrigation (UAI) is contradictory [11–13], it is currently the most commonly used method in clinical practice. It has been demonstrated that UAI increases NaOCl penetration into dentinal tubules [14], and removes accumulated hard tissue debris [15] and bacteria [16] significantly better than syringe irrigation. Anatomically-driven rotary instruments such as the XP-endo Finisher (XPF, FKG Dentaire, Switzerland) can be used to agitate irrigants after root canal preparation. It has been shown to significantly improve the cleaning efficacy of XP-endo Shaper and Reciproc Blue [17].

To the best knowledge of the authors, there is only one study [9] which demonstrated that ultrasonically activated irrigation of NaOCl/HEBP was significantly better than syringe irrigation in eliminating mono-species Enterococcus faecalis from dentinal tubules. However, in the clinical context, biofilms always contain more than one species. In the afore-mentioned study, the role of instrumentation of the root canal to remove biofilms was not taken into account. Furthermore, the effect of different agitation/activation strategies (XPF, UAI) on removal of dual-species biofilms from root canals and dentinal tubules remains unclear. Therefore, the aim of this study was to determine the root canal and dentinal tubule disinfection of NaOCl/HEBP protocol after irrigant agitation/activation with XPF or UAI using a dual-species biofilm model. Syringe-and-needle irrigation (SNI) was used as the control. The null hypothesis was that there was no significant difference between the irrigation protocols or the activation methods in bacterial reduction from the root canals or dentinal tubules.

## Methods

### Sample size calculation

The sample size was calculated based on the data of a previous study [18] by the PASS 11 software (Power Analysis & Sample Size, NCSS, USA). In the ANOVA study, sample size of 12, 12 and 12 were obtained from the 3 groups. The total sample of 36 subjects for experiment groups achieves 87% power to detect differences with a 0.05 significance level.

### Chemicals and reagents

All chemicals and reagents were of reagent grade and purchased from Sigma Aldrich (MO, USA) unless otherwise indicated. Normal saline (0.9% NaCl), NaOCl and EDTA were prepared with deionized water solvent. A pH of 7.5 was maintained for EDTA. A commercially available 0.9 ɡ HEDP powder (Dual Rinse HEDP, Medcem, Vienna, Austria) was freshly mixed with 10 mL 2.5% NaOCl according to the manufacturer’s instructions.

### Specimen selection and preparation

Single-rooted mandibular premolars were obtained by extraction for orthodontic treatment after informed consent of patients and approval of the institutional ethics committee (No. KQEC-2020-63-01). Teeth were stored in 0.9% saline at 4 °C until use. Teeth with cracks, fractures or fused roots were excluded. Radiographs were obtained in the buccolingual and mesiodistal directions to exclude teeth with calcification, multiple root canals or internal resorption. The teeth were decoronated with a diamond disc under copious water cooling. And the root length was further standardized to 13 mm. Teeth with an initial apical file greater than size 15 were excluded. Thirty-six teeth were included in this study. A size 10 K-file was placed in the canal until its tip was visible at the apex, and the working length (WL) was established 1 mm short of this measurement.

### Root canal contamination

Root canal contamination was performed as described previously [9]. Briefly, the root canals were prepared with a size 15 K file using 2.5% NaOCl as the irrigant and 17% EDTA to open the dentinal tubules. This was followed by a final 2.5% NaOCl rinse which was neutralized with 5% sodium thiosulfate. The root canals were then filled with Brain Heart Infusion (BHI) broth using a 30-G needle, and roots were placed into microcentrifuge tubes containing 1 mL BHI and centrifuged twice at 10,000 rpm for 2 min, further transferred to a flask containing 150 mL BHI and sterilized in an autoclave for 15 min at 121 °C. Then, the teeth-containing flask was incubated at 37 °C for 48 h to confirm sterility.

A dual-species biofilm of Enterococcus faecalis (E. faecalis) OG1RF and Streptococcus gordonii (S. gordonii) DL1 was used to infect the root canals. The two species were cultured anaerobically at 37 ℃ (80%N_2_, 10%CO_2_, 10%H_2_) on separated BHI agar plates for 24 h, and a single colony was cultured overnight in BHI broth and then adjusted to 1 × 10^7^ colony-forming units/mL (CFUs/mL). The bacterial suspensions were mixed (1:1) and each canal was filled with 200 μL of the suspension, placed in 2 mL tubes containing BHI broth and incubated anaerobically at 37 °C for 21 days. The culture medium was replenished every other day.

### Verification of established biofilms

Scanning electron microscopy (SEM) was used to confirm if a biofilm was formed throughout the length of the root canal and into dentinal tubules. Two randomly chosen specimens were vertically split through the root canal into 2 halves and fixed in 2.5% glutaraldehyde for 4 h, dehydrated with increasing grades of ethanol, dried using critical point drying and sputter-coated with iridium. The root canal walls and dentinal tubules were observed under SEM (Quanta 400/INCA, FEI, USA).

### Root canal instrumentation and irrigation

**Fig. 1:**
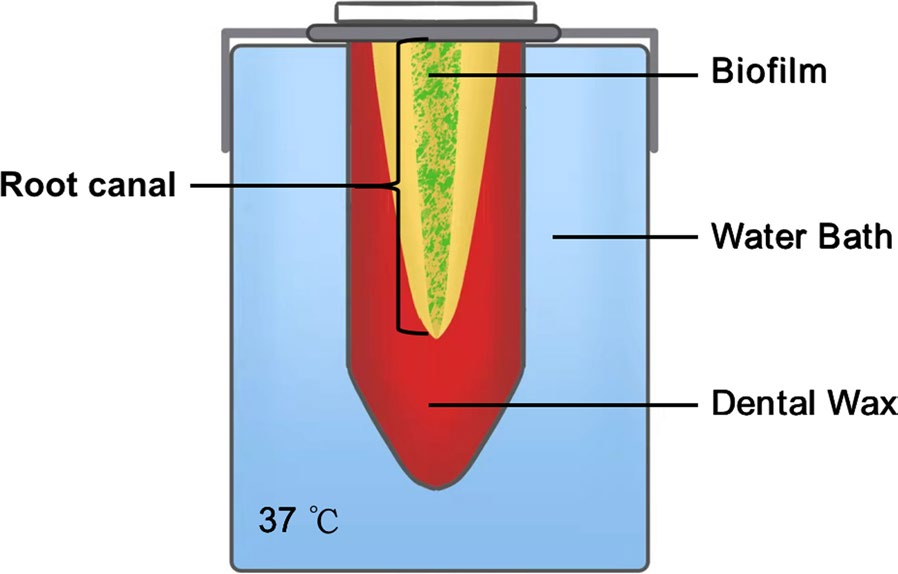
Schematic diagram of the closed-ended root canal system

Thirty-six specimens were randomly distributed into 3 groups (n = 12) based on the final irrigant agitation/activation protocol: group 1, XPF; group 2, UAI and group 3, SNI. A closed-ended root canal system (Fig. 1) was created and the teeth were vertically positioned in a 1.5 mL tube into a water bath at 37 °C to mimic the average intracanal temperature. First, a sample was obtained from the canals before preparation (S1). Canals were rinsed with 1 mL sterile saline for 30 s, and the solution was carried into the whole length of canals with a sterile 15 K-file with gentle circular motions to suspend the bacteria in the canals [19]. Then, three sterile paper points were placed sequentially to the WL to absorb the solution in the canals and transferred to cryotubes containing 1 mL Tris–EDTA (10 mmol/L Tris–HCl, 1 mmol/L EDTA, pH 7.8) buffer. The tube was stored at − 4 °C for 12 h and then frozen at − 20 °C.

Then, a size 15 K-type file was used to confirm patency. Next, the root canals were prepared with a new XP-endo Shaper (XPS; FKG Dentaire, Switzerland) instrument according to the manufacturer’s guidelines. Briefly, canals were irrigated with 2 mL of NaOCl/HEBP and instrumented to working length with XPS for 10 s. Then the canals were rinsed with 2 mL NaOCl/HEBP, and the entire cycle of instrumentation and irrigation was repeated 6 times. The residual NaOCl/HEBP was then absorbed completely by sterile paper points, and canals were rinsed with 1 mL 5% sodium thiosulfate. Then, the sample after preparation (S2) was obtained as described for S1. Following this, the NaOCl/HEBP was agitated/activated as described below.

**Group 1 (XPF)** The canals were flushed with 1 mL NaOCl/HEBP and activated by a new XPF instrument that was placed in the canal to 1 mm short of the working length and powered by the motor at 800 rpm (1 Ncm) for 30 s according to the manufacturer’s instructions. Then the canals were rinsed with 1 mL NaOCl/HEBP for 30 s, and the cycle was repeated once. Finally, the canals were dried and rinsed with 1 mL 5% sodium thiosulfate. The sample after final irrigation (S3) was taken from the canal as described for S1 and S2.

**Group 2 (UAI)** A new ultrasonic tip (K15, Acteon Satelec, France) placed 1 mm short of the working length was activated by an ultrasonic unit (P5 Newtron, Satelec Acteon, Merignac, France) at a power setting of 5 for 30 s, in the presence of 1 mL of NaOCl/HEBP. Then the canals were rinsed for another 30 s with 1 mL NaOCl/HEBP, and the cycle was repeated once. Finally, the canals were dried and rinsed with 1 mL 5% sodium thiosulfate. S3 was taken from the canals as described above.

**Group 3 (SNI)** Root canals were irrigated with 4 mL of NaOCl/HEBP for 2 min using a 30-G side-vented needle placed 1 mm short from the working length. The canals were dried and rinsed with 1 mL 5% sodium thiosulfate. Finally, S3 was taken from the canals as described.

### Quantitative real-time PCR (qPCR)

DNA was extracted from the samples using the TIANamp Bacteria DNA Kit (Tiangen Biotech Co., Ltd., Beijing, China) according to the manufacturer’s instructions. The extracted DNA was used to quantify the amount of E. faecalis and S. gordonii by 16sRNA gene-based qPCR with SYBR Green Premix Pro Taq HS qPCR Kit (Accurate Biotechnology Co., Ltd, Hunan, China) on an ABI QuantStudio 5 real-time PCR instrument (ABI; Thermo Fisher Scientific, Waltham, USA). The total reaction volume was 20 μL. The primers used for E. faecalis and S. gordonii were F: 5′-CGCGAACATTTGATGTGGCT-3′ and R: 5′-GTTGATCCGTCCGCTTGGTA-3′, F: 5′-GCCTTAATAGCACCGCCACT-3′ and R: 5′-CCATCTCTGTTGTTAGGGCGT-3′ respectively [20]. The genomic DNA extracted from a known concentration of E. faecalis OG1RF and S. gordoni DL1 was used to draw a standard curve for quantification. Data were analyzed by ABI 7500 software v2.0.4 (Applied Biosystems) and the specificity of the amplified products was determined by the melting curve analysis.

### Confocal laser scanning microscopy (CLSM)

Specimens were sectioned at 3, 7 and 11 mm from the apical foramen with a diamond disc under water cooling. The root sections were washed gently with sterile saline and stained with 200 μL of LIVE/DEAD BacLight bacterial viability stain (Molecular Probes, Eugene, OR, USA) and incubated in the dark for 15 min. The specimens were visualized under a confocal laser scanning microscope (Carl Zeiss LSM780, Jena, Germany) at the excitation wavelengths of 480/500 nm for SYTO 9 and 490/635 nm for propidium iodide to determine the green and red fluorescence within the dentinal tubules. Three random locations were scanned at each section at a resolution of 1024 × 1024 pixels. Finally, the area ratio of red-to-total fluorescence was calculated to provide the red (apparently dead cells) %, using the Image J program (Image J 1.51v, National Institutes of Health, Bethesda, MD, USA) [21]. Two samples without any treatments were used as negative control.

### Statistical analysis

The normality of the data was determined using Shapiro–Wilk test. Comparison of mean Log_10_CFUs/mL and fluorescence ratios between the groups was analyzed by one-way ANOVA and post-hoc Tukey’s tests. To compare the samples within each group, the paired t-test was used. Statistical Package for Social Science (SPSS, Version 19.0) was used to perform the statistical analysis. The alpha-type error was set at the 5% significance level (α = 0.05).

## Results

**Fig. 2:**
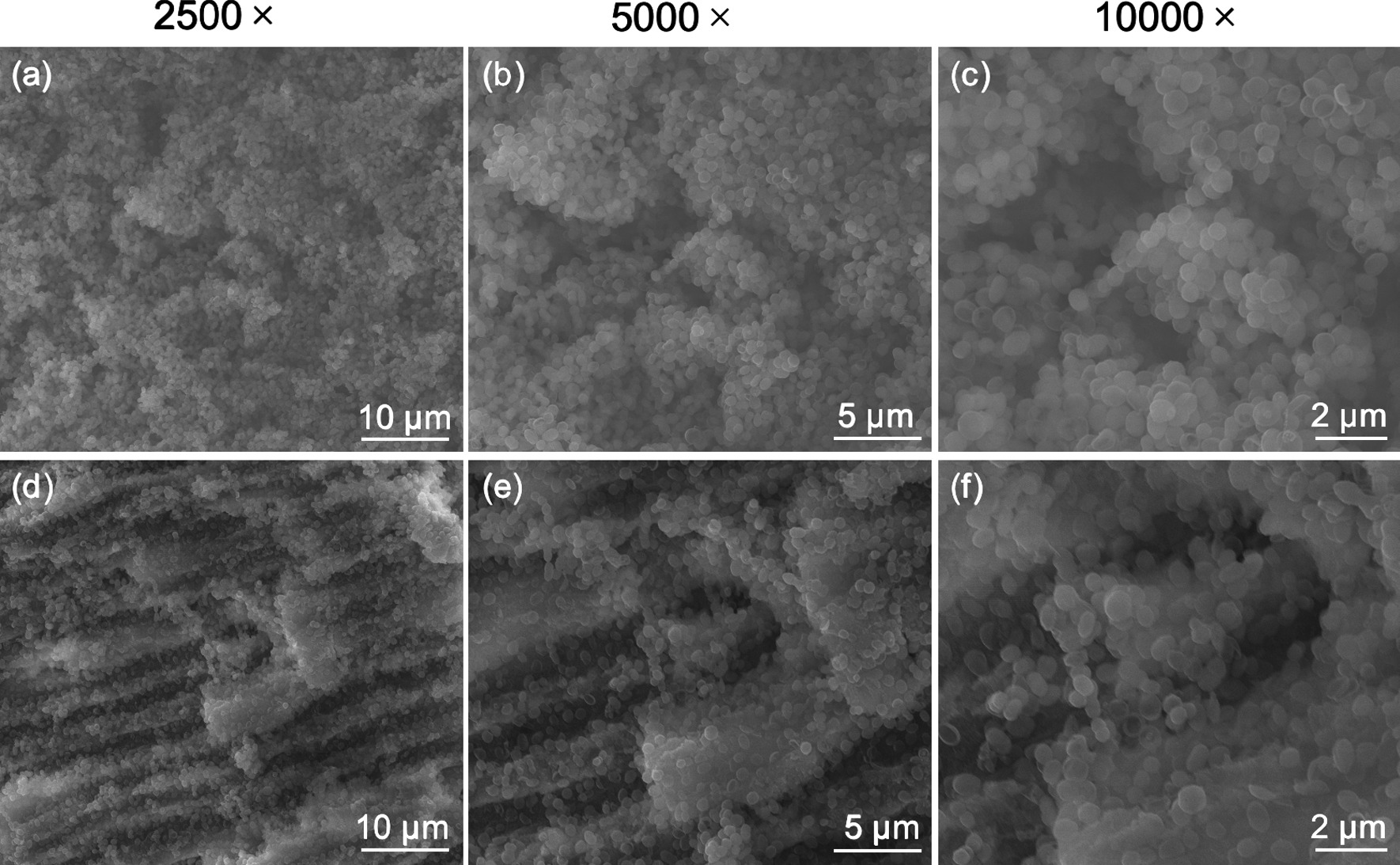
Structure of the 21-day-old dual-species biofilms in root canals. **a**–**c** Bacteria clustered on the root canal wall and covered the root canal dentin completely. **d**–**f** Bacteria also clustered into the dentinal tubules

The SEM images validated the formation of a dense biofilm, with large bacterial clusters covering the root dentin (Fig. 2a–c) and penetrating into the dentinal tubules (Fig. 2d–f).

**Fig. 3:**
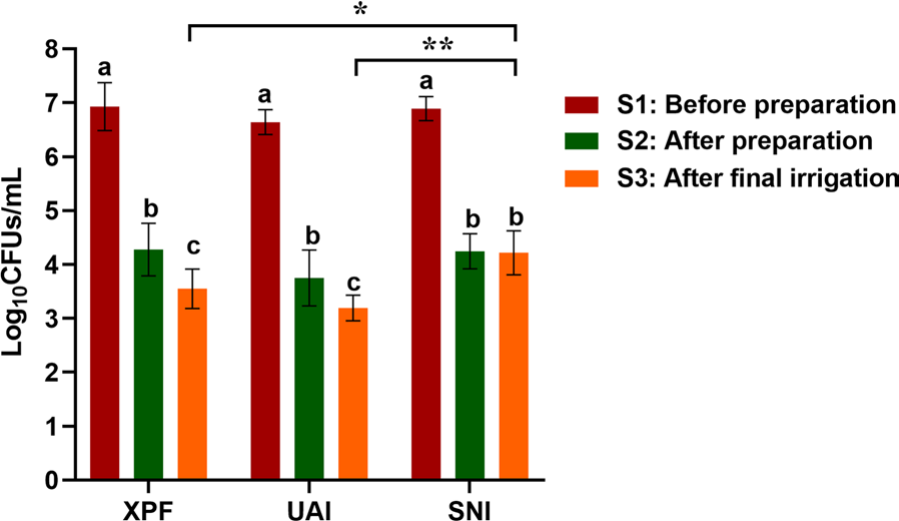
Bacterial counts before preparation (S1), after preparation (S2) and after final irrigation (S3). Different lower-case letters indicate significant differences within each group (P < 0.05). Asterisks indicate significant differences of S3 among groups (*P < 0.05, **P < 0.01)

The reduction of bacterial counts (log_10_CFUs/mL) was assessed by qPCR before preparation (S1), after preparation (S2) and after final irrigation (S3) (Fig. 3). There was no significant difference (P > 0.05) between the groups in terms of bacteria counts at S1 and S2. However, comparing the S3 counts between the three groups, no significant difference was observed between XPF and UAI (P > 0.05), both of them showed significantly less bacterial counts than SNI (P < 0.05). For all groups, there was a significant reduction in bacteria counts from S1 to S2 (P < 0.0001). However, the S3 bacterial counts was significantly less than the S2 bacterial counts only in the XPF and UAI treated groups(P < 0.05). A Additional file 1 shows the bacterial counts of E. faecalis and S.gordonni separated in more detail [see Additional file 1].

**Fig. 4:**
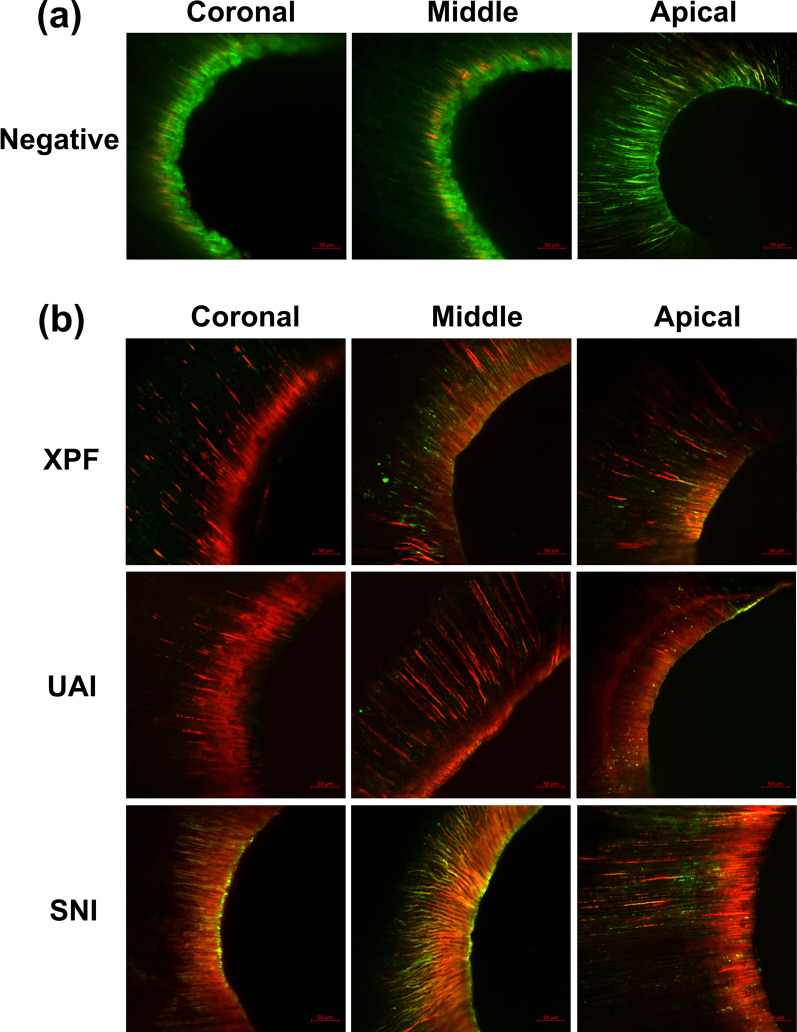
CLSM images showing green-stained (apparently live) and red-stained (apparently dead) bacteria within dentinal tubules. **a** The merged images from negative control samples. **b** Representative merged images from different experimental groups. The scale bars represent 50 μm

Figure 4 shows the CLSM images of live and dead bacteria within the dentinal tubules. The mean (± SD) red to total fluorescence indicating the dead cells within dentinal tubules has been shown in Table 1. XPF and UAI showed significantly greater percentage of dead bacteria within the dentinal tubules than syringe-and-needle irrigation in the coronal-third (P < 0.05); UAI showed significantly greater percentage of dead bacteria within the dentinal tubules in the middle-third (P < 0.05); there was no difference between the three groups in the apical-third (P > 0.05).

**Table 1:** The ratio (Mean ± SD) of red to red-green combined fluorescence area in different groups and regions

Group	N	Coronal region	Middle region	Apical region
XPF	12	0.74 ± 0.15^a^	0.62 ± 0.06^a^	0.63 ± 0.07^a^
UAI	12	0.75 ± 0.08^a^	0.76 ± 0.08^b^	0.71 ± 0.13^a^
SNI	12	0.58 ± 0.07^b^	0.63 ± 0.11^a^	0.64 ± 0.09^a^

Different lower-case letters indicate significant differences between groups in the same region (P < 0.05)

## Discussion

The present study aimed at comparing the effect of a rotary instrument-based irrigant agitation strategy (XP-endo Finisher) with a commonly used irrigant activation strategy (ultrasonic) on the removal of dual-species biofilms from root canals and bacterial killing within dentinal tubules, after root canal instrumentation with an anatomically conforming coreless instrument (XP-endo Shaper). It has been shown that mixed-species biofilms display higher resistance to intracanal treatment procedures [22]. It was known that endodontic infections were caused by multiple species [23, 24]. E. faecalis has been reported to be more prevalent in persistent endodontic infections in comparison with other bacterial species, and survive in the undernourished environment of root canal through interspecies interactions [23–25]. Some streptococci, such as S. gordonii, have also been identified prevalently in persistent endodontic infections[26, 27]. Moreover, the combination of E. faecalis and S. gordonii have been shown to form dense and thick biofilms on root canal dentin [25]. Consistently, in this proof-of-principle study, a dense dual-species biofilm was formed after incubating with E. faecalis and S. gordonii for 21 days, attempting to mimic the infected status within the root canal as far as possible. Nevertheless, this present biofilm model has its own limitation, which may not sufficiently represent the complexity of the diverse microbiota in the root canal. Hence, multi-species biofilm model can be taken into consideration in further research.

The irrigation regimen used in this study was a mixture of sodium hypochlorite with etidronate i.e., continuous chelation. It has been shown in vitro that this approach results in superior root canal disinfection compared to the traditional NaOCl/EDTA irrigation [9], without the harsh effects on dentine imposed by the latter [7]. One previous study used a mono-species biofilm model to demonstrate that laser activation of NaOCl/HEBP resulted in superior canal disinfection compared to ultrasonically activated irrigation [9]. However, lasers are not commonly used in clinical practice and it is essential to demonstrate if currently available, novel instrumentation and agitation approaches can improve the disinfection potential of NaOCl/HEBP. Therefore, root canals were instrumented with XPS, using NaOCl/HEBP as the irrigant.

The XPS is composed of the MaxWire alloy and has been shown to change shape with changes in temperature [28]. It is in the martensitic phase at room temperature, then transforms to the austenitic phase with its characteristic "snake" shape at body temperature. This shape change allows for superior contact with the dentin walls and achieves a final preparation of about 30/0.04 [29].

The results of this study showed that instrumentation with XPS using NaOCl/HEBP significantly reduced the bacterial burden from root canals. Notably, it could remove more than 99% of the bacteria from root canals. This corroborates with well-established findings that instrumentation alone can remove a substantial part of the septic content from root canals [30]. Such findings for XPS are in agreement with a previous study [31]. Notably, the afore-mentioned study used only saline as the irrigation solution and yet, 80.3% reduction of E. faecalis was achieved with XPS. In the current study, NaOCl/HEDP was used as the irrigant during the instrumentation with XPS and more bacterial reduction (> 99%) was achieved. Therefore, it may be assumed that XPS works synergistically with disinfectants to achieve superior bacterial reduction.

The main outcome variable in this work was the bacterial counts within the root canal and dead bacterial percentage within the dentinal tubules, when NaOCl/HEDP was subject to XPF agitation, UAI or syringe irrigation. The results showed that there was no significant difference between XPF and UAI in the bacterial reduction from root canals. Such results have also been demonstrated in a clinical trial where UAI and XPF agitation of NaOCl showed no difference between each other in bacterial reduction, but both were significantly superior to syringe irrigation [16]. While the microbial reduction with UAI may be attributed to the cavitation and acoustic streaming caused by ultrasonic waves [32] or the increased temperature of the irrigants [33] which is conductive to the enhanced effectiveness of the irrigants and the removal of bacterial biofilm from root canal system [34], microbial reduction with XPF agitation may be attributed to its the “spoon-like” shape, which has been claimed to enhance its adaptation to the canal walls and cause powerful irrigant streaming to detach the microbes from the infected root canals [35].

CLSM analysis showed that the percentage of dead bacteria within dentinal tubules ranged from 58 to 76%, indicating that none of the methods used in this work completely eliminated bacteria from the dentinal tubules. As expected, syringe-and-needle irrigation resulted in the least percentage of dead bacteria within the dentinal tubules in the coronal-third. The use of XPF and UAI promotes irrigant exchange and improves the flow dynamics of the irrigant across all regions of the canal [36, 37]. Previous study reported that ultrasonic irrigation led to deeper penetration of irrigants than needle irrigation [38] which contributed to the improved disinfection of dentinal tubules. In addition, the mechanical effect and irrigant agitation of XPF with high-speed rotation may enhance the detaching of biofilm from the canal wall. A notable finding was that there was no significant difference between UAI, XPF and syringe-and-needle irrigation in the dead bacterial percentage within dentinal tubules in the apical third, while UAI resulted in significantly greater bacterial killing within dentinal tubules in the middle-third. It is possible that ultrasonic activation did not have sufficient energy to generate the acoustic streaming and hydrodynamic shear stress propagation in the apical third. It has been shown considerable file-to-wall contact occurred during ultrasonic activation. The oscillation of the ultrasonic file was affected by the contact with the root canal wall, while the apical third was the most affected among the different parts of root canal in the size 35/0.06 or 50/0.06 [39]. A reduction of approximately 35% in the oscillation amplitude was noticed in all contact cases compared to the non-contact cases, though the file oscillation was not dampened completely during the wall contact [39]. Accordingly, in the present study, UAI in the apical third failed to achieve the equal efficacy to that in the middle third, leading to no significant difference of dead bacterial percentage between UAI, XPF and SNI in apical third.

Indeed, one may question the rationale behind not including the traditional NaOCl/EDTA protocol in the experimental design. However, the focus of this work was to investigate the methods to enhance disinfection using the NaOCl/HEBP protocol with the instruments that are currently available. Comparing the NaOCl/EDTA and NaOCl/HEBP protocols in the current study design is challenging in terms of standardization of duration and volume as the former is a sequential regimen and the latter is a combined regimen. Furthermore, it has been reported that the NaOCl/HEBP protocol is superior to the NaOCl/EDTA protocol in eliminating biofilms from root canals [9, 40]. Previous study showed that NaOCl/HEBP, regardless of being activated by diode laser, Er:YAG laser or ultrasonic, was capable to cause significantly greater reduction of E. faecalis biofilm in the root canal lumen, compared to NaOCl/EDTA [9]. Likewise, Giardino L, et al. [40] found that the all-in-one irrigant NaOCl/HEBP resulted in significantly lower residual bacterial viability (1.71%) of E. faecalis in dentinal tubules than the NaOCl/EDTA sequential irrigarion (3.77%). One important limitation in this work was the sampling approach. Although paper points are a well-established and commonly used method [23], it obtains samples only from the main canal and not the microanatomy. There are some alternative approaches, such as cryogrinding [41] where sampling can be obtained from the entire tooth, or observing the biofilms directly at high magnifications [42]. But both of them are destructive and the bacterial samples can only be collected for one time. The present study did not employ cryogrinding since the samples were taken by paper points and observed the bacteria viability under CLSM to make up for the limitation.

## Conclusions

The results of study highlight that XP-endo Finisher and ultrasonically activated irrigation are equally effective but significantly more effective than syringe irrigation in disinfecting the main canal space and dentinal tubules in the coronal third when a mixture of NaOCl/HEBP is used for irrigation, while ultrasonically activated irrigation displayed better disinfection within dentinal tubules in the middle third.

## Supplementary Information

**Additional file 1**. Bacterial counts of* E. faecalis* (a) and S.gordonni (b) before preparation (S1), after preparation (S2) and after final irrigation (S3).

## Data Availability

The datasets generated and analyzed during the current study are not publicly available due to data privacy and security, but are available from the corresponding author upon reasonable request.

## Abbreviations

BHI: Brain Heart Infusion
CFU: Colony-forming units
CLSM: Confocal laser scanning microscopy
EDTA: Ethylenediaminetetraacetic acid
E. faecalis: Enterococcus faecalis
HEBP: Etidronate, 1-hydroxyethylidene-1,1-bisphosphonate
NaOCl: Sodium hypochlorite
qPCR: Quantitative real-time PCR
UAI: Ultrasonically activated irrigation
SEM: Scanning electron microscopy
S. gordonii: Streptococcus gordonii
SNI: Syringe-and-needle irrigation
XPF: XP-endo Finisher
XPS: XP-endo Shaper
