# Supplementary material for: Impact of agitation/activation strategies on the antibiofilm potential of sodium hypochlorite/etidronate mixture in vitro
Source: BMC Oral Health. 2022 May 23;22:201. doi: 10.1186/s12903-022-02222-1 (PMC9125931; doi:10.1186/s12903-022-02222-1)
Supplement: Supplementary file 1 — Additional file 1. Bacterial counts of E. faecalis (a) and S.gordonni (b) before preparation (S1), after preparation (S2) and after final irrigation (S3). [file 12903_2022_2222_MOESM1_ESM.pdf]

Supplementary material. Bacterial counts of E. faecalis (a) and S.gordonni (b) before preparation (S1), after preparation (S2) and after final irrigation (S3).

(a)

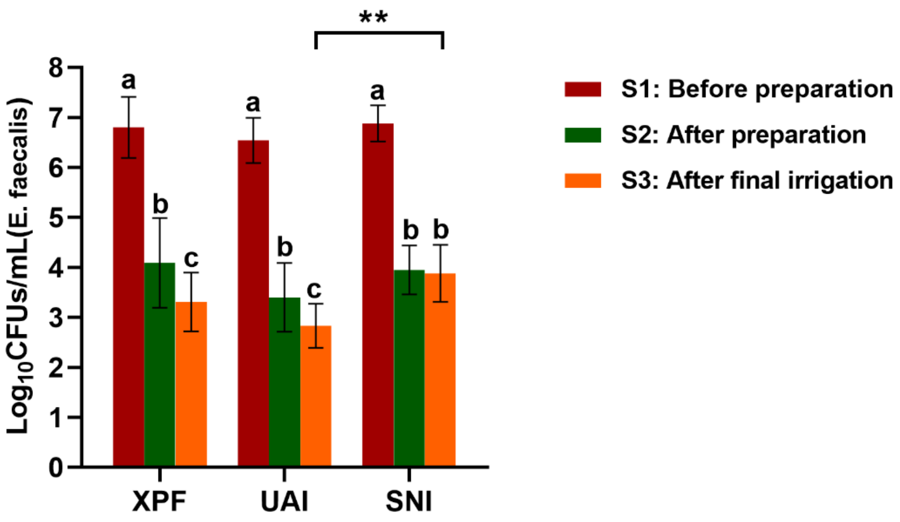

(b)

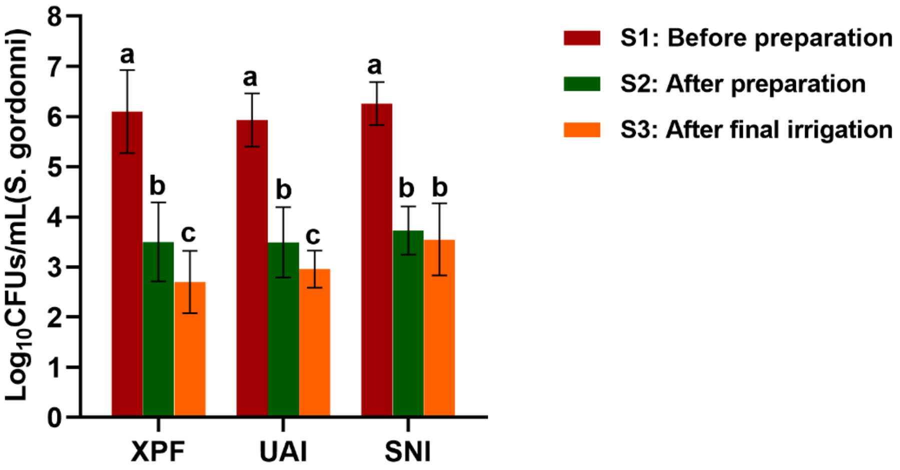

Different lower-case letters indicate significant differences within each group ($P<0.05$).

Asterisks indicate significant differences of S3 among groups (**$P<0.01$).
